# First-Line Ipilimumab with Nivolumab versus Immune Checkpoint Inhibitors with Tyrosine Kinase Inhibitors in Patients with Intermediate- or Poor-Risk Metastatic Clear Cell Renal Cell Carcinoma

**DOI:** 10.15586/jkc.v12i2.387

**Published:** 2025-04-25

**Authors:** Micah Ostrowski, Yeonjung Jo, Georges Gebrael, Chadi Hage Chehade, Zeynep Irem Ozay, Blake Nordblad, Ayana Srivastava, Diya Garg, Richard Ji, Gliceida Galarza Fortuna, Vinay Mathew Thomas, Beverly Chigarira, Ethan Anderson, Neeraj Agarwal, Benjamin L. Maughan, Umang Swami

**Affiliations:** 1 Division of Medical Oncology, Department of Internal Medicine, Huntsman Cancer Institute, University of Utah, Salt Lake City, UT, USA;; 2 Cancer Biostatistics, Huntsman Cancer Institute, University of Utah, Salt Lake City, UT, USA

**Keywords:** immune checkpoint inhibitors, metastasis, renal cell carcinoma, survival outcomes, targeted therapy

## Abstract

Ipilimumab with nivolumab (Ipi + Nivo) and immune checkpoint inhibitors with tyrosine kinase inhibitors (ICI + TKI) are the first-line approved treatments for intermediate- and poor-risk metastatic clear cell renal cell carcinoma (mccRCC); however, they have not been compared head-to-head in prospective trials to guide treatment selection. Thereupon, we sought to compare survival outcomes of patients receiving first-line Ipi + Nivo versus ICI + TKI, using a large, real-world database among patients with intermediate- and poor-risk mccRCC. This retrospective cohort study used a nationwide electronic health record-derived deidentified database, where patients with mccRCC with intermediate- or poor-risk who received first-line Ipi + Nivo or ICI + TKI between 20 June, 2016, and 26 January, 2023, were included. Primary outcomes were real-world time to next therapy (rwTTNT) and real-world overall survival (rwOS), summarized via Kaplan-Meier survival estimates with 95% confidence intervals (CIs) and compared in the context of propensity score (PS) matching weighted analysis. Of the 12,707 patients in the dataset, 1,438 with mccRCC met eligibility and were included. After PS matching weighted analysis, no significant difference in rwOS was noted between both groups (HR 1.01, 95% CI 0.86–1.19; p = 0.91); however, rwTTNT was significantly shorter with Ipi + Nivo than with ICI + TKI (HR 0.78, 95% CI 0.68–0.89; p < 0.001). In this large real-world study, there was evidence that rwOS was comparable, while rwTTNT was superior in patients receiving ICI + TKI compared to those receiving Ipi + Nivo. These real-world data offer important guidance for clinicians in choosing between Ipi + Nivo and ICI + TKI as frontline treatment.

## Introduction

The treatment landscape of metastatic clear cell renal cell carcinoma (mccRCC) has undergone significant evolution over the past decade. Currently, immune checkpoint inhibitor (ICI)-based combinations are the standard of care in the first-line setting. According to the recent National Comprehensive Cancer Network (NCCN) guidelines, approved regimens include dual ICIs such as ipilimumab with nivolumab (Ipi + Nivo) or ICIs combined with vascular endothelial growth factor (VEGF) tyrosine kinase inhibitors (TKIs) such as cabozantinib with nivolumab, pembrolizumab with axitinib, lenvatinib with pembrolizumab, and axitinib with avelumab (1). These combinations were investigated in randomized controlled trials, all demonstrating superiority over sunitinib monotherapy (2–6).

Based on the CheckMate-214 trial, Ipi + Nivo became the first US Food and Drug Administration (FDA)-approved dual ICI combination in 2018 for patients in the International Metastatic Renal Cell Carcinoma Database Consortium’s (IMDC’s) intermediate- and poor-risk subgroups (2). The initial trial report demonstrated a 37% reduction in the risk of death with the combination therapy compared to sunitinib alone; however, in patients with a favorable IMDC risk score, the overall survival (OS) data favored sunitinib. In the final survival analysis of the trial conducted with an 8-year follow-up, the median OS in the combination arm reached 52.7 months compared to 37.8 months in the sunitinib arm (hazard ratio [HR] 0.72, 95% confidence interval [CI], 0.62–0.83) (7). Additionally, in the favorable IMDC risk group, though not statistically significant, there was a trend favoring the combination arm, with a median OS of 77.9 months versus 66.7 months (HR 0.82, 95% CI 0.6–1.13). In response to these findings, the NCCN guidelines were amended to recommend Ipi + Nivo for favorable risk as well as for intermediate- and poor-risk disease (1).

Similarly, pembrolizumab with axitinib was the first ICI and TKI combination approved for the treatment of metastatic renal cell carcinoma (mRCC), based on the Keynote-426 trial (6). In this trial, patients across all IMDC risk scores benefited from the combination, with a 47% reduction in the risk of death observed. At the 43-month follow-up, the combination continued to be associated with improved OS with an HR of 0.73 (8).

Following pembrolizumab and axitinib, additional ICI + TKI combinations, including cabozantinib with nivolumab, lenvatinib with pembrolizumab, and axitinib with avelumab, gained approval for the treatment of mRCC. In the Checkmate 9ER trial, patients treated with cabozantinib with nivolumab had a superior progression-free survival (PFS) compared to those treated with sunitinib (4). In the final survival analysis, with a median follow-up of 67.6 months, sustained benefit with cabozantinib and nivolumab was demonstrated in median PFS (HR 0.58, 95% CI 0.49–0.70) and OS (HR 0.79, 95% CI 0.65–0.96) (9). Similarly, the JAVELIN Renal 101 study demonstrated better PFS in patients treated with axitinib with avelumab compared to sunitinib (5). In the CLEAR trial, patients across all IMDC risk groups treated with lenvatinib with pembrolizumab were proven to have a 34% reduction in the risk of death compared to single agent sunitinib (3).

However, in the absence of head-to-head comparisons between dual ICI compared to ICI + TKI and validated biomarkers to guide treatment decisions, the selection of a first-line regimen remains heavily dependent on factors such as the IMDC risk criteria, physician’s preference, patient comorbidities, disease burden, and financial considerations, among others. Thus, real-world data remains an important resource to guide clinicians in choosing between these first-line regimens and helping in patient counseling. In this study, we utilized a large, real-world database to directly compare survival outcomes of patients treated with first-line Ipi + Nivo versus ICI + TKI in patients with intermediate- or poor-risk mccRCC.

## Materials and Methods

### Cohorts and exposures

This retrospective cohort study utilized the nationwide Flatiron Health’s electronic health records (EHRs)-derived database—a longitudinal, real-world database—comprising deidentified patient-level data, structured and unstructured, originating from approximately 280 cancer clinics representing roughly 800 sites of care across the United States and curated via technology-enabled abstraction (10). The data are subject to obligations to prevent reidentification and protect patient confidentiality. This study includes patients diagnosed with mccRCC and treated with systemic therapies in the United States and was approved by the Institutional Review Board at the University of Utah.

The initial cohort consisted of patients diagnosed with mccRCC from January 11, 2011, to January 31, 2023, receiving first-line therapy from June 20, 2016, to January 26, 2023. Baseline characteristics, including age, race, ethnicity, smoking status, insurance, practice type, prior nephrectomy, and the year of first-line therapy initiation were collected on the same date of the initiation. All patients had intermediate or poor IMDC risk scores based on six prognostic variables, including Karnofsky Performance Status, time from diagnosis to first-line systemic therapy, hemoglobin level, corrected calcium, absolute neutrophil count, and platelet count. Patients were assigned to poor risk if they possessed at least three negative prognostic factors. Intermediate risk was assigned to patients with one or two negative prognostic factors and no missing values or to patients with one negative prognostic factor and zero or one missing value. Poor/intermediate risk was assigned to patients with one or two negative prognostic factors but with two or more missing values. Patients with zero negative prognostic factors with or without missing values were excluded from the analysis.

### Outcomes and analyses

The primary endpoints were real-world overall survival (rwOS) and real-world time to next therapy (rwTTNT), with the index date defined as the date of first-line therapy initiation. rwOS was defined as the number of months from the index date to the date of death or loss to follow-up and rwTTNT was defined as the number of months from the index date to the date of second-line therapy initiation, death, or loss to follow-up. Patients were censored at their last engagement date in structured activities, which included visits, drug episodes, and oncology expert-defined, rules-based line of therapy records in patients’ EHRs.

For insurance status, missing start or end dates were imputed assuming the calendar year coverage. Any missing values in baseline characteristics were multiply imputed using predictive mean matching on 50 chained equations, including all the baseline characteristics and outcomes in the equations (11). The outcomes were summarized using Kaplan-Meier survival estimates with 95% CIs. The propensity score (PS) was estimated using logistic regression for treatment at the first-line conditioning on the missing data in baseline covariates, allowing for restricted cubic splines with three knots for continuous covariates to allow nonlinear predictors of treatment. Baseline covariates included age, race, ethnicity, smoking status, insurance, practice type, prior nephrectomy, IMDC risk score, and the year of first-line therapy initiation. Matching weights were used to reweigh the analytic cohort (12). Balance in baseline characteristics by the first-line treatment was assessed using the standardized mean difference (SMD) and rechecked after reweighing. We fitted marginal Cox proportional hazards models comparing treatment at the first line (Ipi + Nivo with ICI + TKI) on rwOS and rwTTNT on a reweighted analytic cohort. As sensitivity analyses, we fitted adjusted Cox proportional hazards models adjusting for baseline covariates, except for the year of first-line initiation. The proportional hazards assumption was checked using Schoenfeld’s residuals (13). We calculated the median follow-up time using the reverse Kaplan-Meier method. All analyses were done using the R version 4.2.3 and a multivariate imputation by chained equations package was used for multiple imputations (14, 15).

## Results

**Figure 1:**
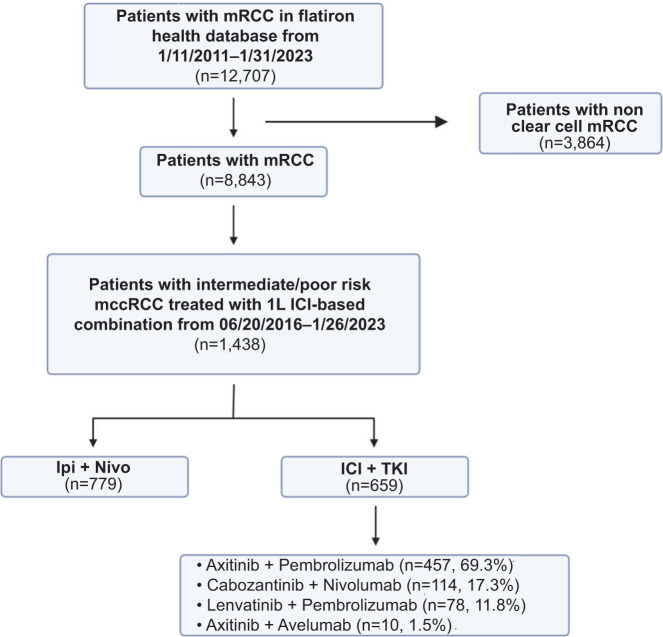
STROBE flowchart of patient selection. Metastatic renal cell carcinoma (mRCC), metastatic clear cell renal cell carcinoma (mccRCC), first-line (1L), immune checkpoint inhibitor (ICI), ipilimumab + nivolumab (Ipi + Nivo), tyrosine kinase inhibitor (TKI).

The initial cohort comprised 12,707 patients with renal cell carcinoma; the final analytic cohort consisted of 1,438 patients with clear cell histology and intermediate/poor risk who received at least one line of approved systemic therapy per NCCN guidelines with available OS. Treatment received included Ipi + Nivo (n = 779), pembrolizumab with axitinib (n = 457), nivolumab with cabozantinib (n = 114), pembrolizumab with lenvatinib (n = 78), and avelumab with axitinib (n = 10). A STROBE flow diagram is presented in Figure 1. Patients were classified into two groups based on whether they were treated with dual ICI combination of Ipi + Nivo (n = 779; 54%) or with ICI and TKI combinations (n = 659; 46%) such as pembrolizumab with axitinib, nivolumab with cabozantinib, pembrolizumab with lenvatinib, and avelumab with axitinib.

Baseline characteristics of patients receiving Ipi + Nivo and ICI + TKI are summarized in Table 1. Race, smoking status, insurance, IMDC risk group, and prior nephrectomy were well balanced, with the SMD being less than 0.1. Age, practice type, and the start year of treatment showed imbalances (SMD > 0.1), with patients in the ICI + TKI group being slightly older (65 versus 67 years) and having initiated therapy later. All the baseline covariates achieved adequate balance (SMD < 0.1) following propensity score matching weighted analysis.

**Table 1:** Baseline characteristics of included patients.

Baseline Characteristics	Ipi + Nivo (n = 779)	ICI + TKI (n = 659)	SMD	Missing (%)
Age,^a^ mean (SD)	65.03 (10.61)	66.77 (10.32)	0.166	0.0
Race/ethnicity, n (%)				
Asian non-Hispanic	16 (2.3)	13 (2.2)	0.072	10.6
Black non-Hispanic	45 (6.5)	30 (5.1)		
Hispanic/Latino	66 (9.5)	50 (8.5)		
White non-Hispanic	490 (70.4)	426 (72.3)		
Other^b^	79 (11.4)	70 (11.9)		
Smoking Status, n (%)				
No history of smoking	333 (42.9)	277 (42.1)	0.015	0.2
History of smoking	444 (57.1)	381 (57.9)		
Practice Type, n (%)				
Community	635 (81.5)	569 (86.3)	0.132	0.0
Academic	144 (18.5)	90 (13.7)		
Insurance, n (%)				
Commercial health plan	500 (72.8)	458 (76.3)	0.092	10.5
Medicare/other government program	103 (15.0)	81 (13.5)		
Medicaid	18 (2.6)	16 (2.7)		
Other	66 (9.6)	45 (7.5)		
Prior Nephrectomy, n (%)				
Yes	398 (51.2)	334 (50.8)	0.008	0.1
No	380 (48.8)	324 (49.2)		
Performance Status, n (%)				
ECOG ≥ 2	103 (13.2)	123 (18.7)	0.26	0.0
ECOG < 2	526 (67.5)	465 (70.6)		
Unknown	150 (19.3)	71 (10.7)		
First-Line Start Year, n (%)				
2016	2 (0.3)	0 (0.0)	0.758	0.0
2017	4 (0.5)	2 (0.3)		
2018	138 (17.7)	1 (0.2)		
2019	175 (22.5)	109 (16.5)		
2020	154 (19.8)	128 (19.4)		
2021	173 (22.2)	212 (32.2)		
2022	131 (16.8)	193 (29.3)		
2023	2 (0.3)	14 (2.1)		
IMDC Risk Group, n (%)				
Intermediate risk	368 (47.2)	325 (49.3)	0.052	0.0
Poor risk	186 (23.9)	144 (21.9)		
Poor/intermediate risk	225 (28.9)	190 (28.8)		

a Patients with a birthyear of (data cutoff year: 85) or earlier may have an adjusted Birthyear in Flatiron datasets due to patient deidentification requirements.

b Alaska Native, American Indian, Native Hawaiian, other Pacific Islanders who are not Hispanic or Latino or a race description which falls in multiple race categories.

Standard deviation (SD), standardized mean difference (SMD), ipilimumab + nivolumab (Ipi + Nivo), immunotherapy + tyrosine kinase inhibitor (ICI + TKI), Eastern Cooperative Oncology Group (ECOG), International Metastatic Renal Cell Carcinoma Database Consortium (IMDC).

**Figure 2:**
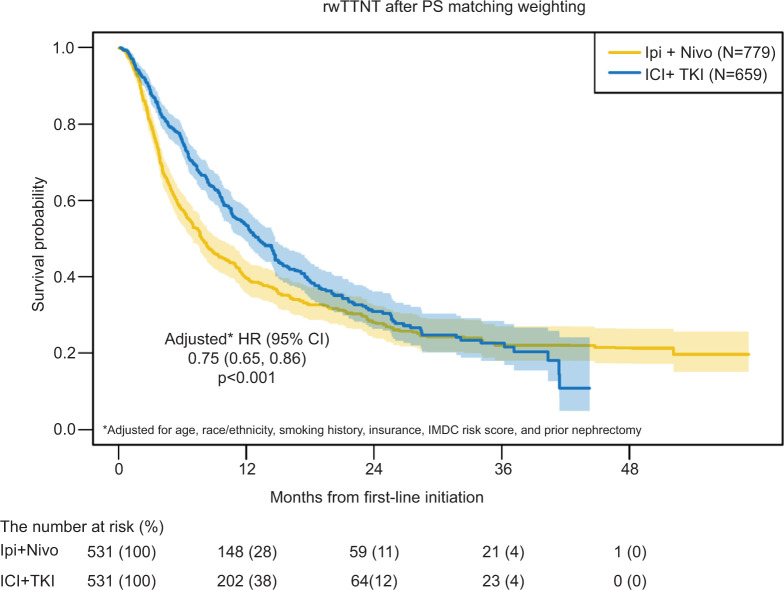
Real-world TTNT from first-line after propensity score matching weighted analysis. rwTTNT summarized via Kaplan-Meier survival estimates with 95% CI and compared in the context of propensity score matching weighted analysis. After adjusting for age, race/ethnicity, smoking history, IMDC risk score, and prior nephrectomy, rwTTNT was significantly shorter in patients treated with Ipi + Nivo than those treated with ICI + TKI (HR 0.75, 95% CI 0.65–0.86; p < 0.001). Real-world time to next therapy (rwTTNT), propensity score (PS), ipilimumab + nivolumab (Ipi + Nivo), immunotherapy + tyrosine kinase inhibitor (ICI + TKI).

The median rwTTNT was 8.3 months (95% CI 7.6–10) for patients treated with Ipi + Nivo (reference group) and 13 months (95% CI 12–15) for patients treated with ICI and TKI. The rwTTNT hazard ratio for ICI + TKI compared to ipilimumab and nivolumab was 0.78 (95% CI 0.68–0.90; p < 0.001). After PS matching weighted analysis, median rwTTNT was 7.8 months (95% CI 6.9–9.4) for patients treated with ipilimumab and nivolumab compared to 13.1 months (95% CI 11.8–14.7) for patients treated with ICI and TKI (Figure 2). The rwTTNT HR for ICI + TKI compared to the Ipi + Nivo group after PS matching weighted analysis was 0.78 (95% CI 0.68–0.89; p < 0.001). The adjusted model yielded the same conclusion, with an HR of 0.75 (95% CI 0.65–0.86; p < 0.001).

**Figure 3:**
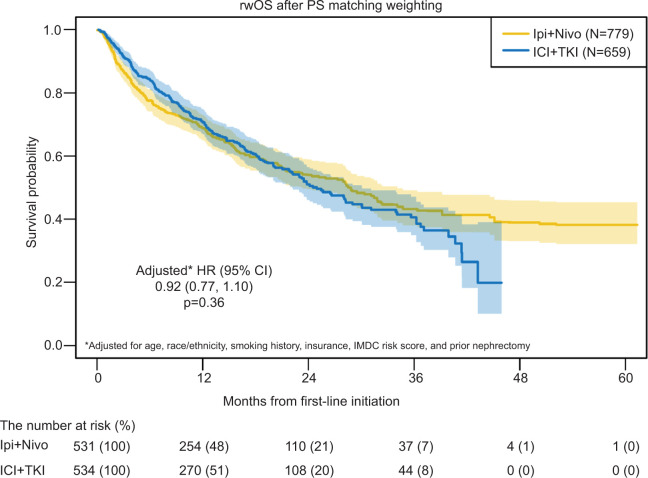
Real-world OS from first-line after propensity score matching weighted analysis. rwOS summarized via Kaplan-Meier survival estimates with 95% CI and compared in the context of propensity score matching weighted analysis. After adjusting for age, race/ethnicity, smoking history, IMDC risk score, and prior nephrectomy, there was evidence that rwOS was similar between Ipi + Nivo and ICI + TKI groups (HR 0.92, 95% CI 0.77–1.10; p = 0.36). Real-world overall survival (rwOS), propensity score (PS), ipilimumab + nivolumab (Ipi + Nivo), immunotherapy + tyrosine kinase inhibitor (ICI + TKI).

The median rwOS for patients receiving Ipi + Nivo (reference group) was 28 months (95% CI 23–31) compared to 25 months (95% CI 22–30) for those treated with ICI and TKI (HR 1.01, 95% CI 0.86–1.19; p = 0.86). After PS matching weighted analysis, the median rwOS was 28.8 months (95% CI 23.3–34.1) for patients treated with Ipi + Nivo compared to 25 months (95% CI 22.1–30.1) for patients treated with ICI and TKI (Figure 3). After PS matching weighted analysis, there was no evidence that the rwOS was different between the Ipi + Nivo and ICI + TKI groups (HR 1.01, 95% CI 0.86–1.19; p = 0.91). The adjusted model yielded the same conclusion (HR 0.92, 95% CI 0.77–1.10; p = 0.36). Among the individual ICI + TKI regimens, the median rwOS was 25 months (95% CI 12–not reached [NR]) for avelumab + axitinib, 25 months (95% CI 22–34) for axitinib + pembrolizumab (Pembro + Axi), 22 months (95% CI 20–NR) for cabozantinib + nivolumab, and 17 months (95% CI 11–NR) for lenvatinib + pembrolizumab. The median follow-up time was 21.16 months in the overall cohort, 25.89 for the Ipi + Nivo group, and 16.99 months for the ICI + TKI group.

Of patients receiving first-line Ipi + Nivo, 313 (40.2%) received second-line therapy. The most common second-line therapies in this cohort were TKI monotherapy (178 patients [22.8%]), ICI + TKI (76 patients [9.8%]), and ICI monotherapy (16 patients [2.1%]). Of patients receiving first-line ICI + TKI, 184 (27.9%) received second-line therapy. In this cohort, TKI monotherapy was the most common (88 patients [13.4%]), followed by ICI + TKI (25 [3.8%]) and Ipi + Nivo (23 patients [3.5%]).

## Discussion

In this study, we observed that patients with intermediate- or poor-risk mccRCC treated with ICI and TKI had significantly better rwTTNT than those treated with Ipi + Nivo, while rwOS was comparable between the two groups. rwOS was similar among the four individual ICI + TKI regimens. To our knowledge, this is the largest real-world study comparing outcomes of Ipi + Nivo and ICI + TKI as first-line treatment in a homogeneous population of patients with mccRCC with intermediate or poor IMDC risk.

Our results are concordant with retrospective studies comparing real-world outcomes between Ipi + Nivo and ICI + TKI. In one study, 331 patients with mRCC were treated with Ipi + Nivo or Pembro + Axi, and rwTTNT was significantly longer in patients receiving Pembro + Axi (16). Additionally, real-world progression-free survival (rwPFS) and real-world treatment time were significantly higher in the group receiving Pembro + Axi. Similarly, a real-world analysis comparing 1506 patients with mRCC treated with Ipi + Nivo or Pembro + Axi demonstrated better median rwPFS in patients treated with Pembro + Axi (17). Median OS was superior in the Pembro + Axi group for those with IMDC favorable-risk disease. However, there was no difference in median OS in intermediate- or poor-risk groups.

In addition to real-world studies, network meta-analyses (NMAs) have compared outcomes between Ipi + Nivo and ICI + TKI. Although these NMAs are limited by the heterogeneity of the trials from which they are comprised, they are consistent with real-world retrospective studies showing favorable outcomes with ICI + TKI compared to Ipi + Nivo. Quahal et al. conducted an NMA using data from six phase III randomized control trials to compare PFS and OS between Ipi + Nivo, ICI + TKI, and atezolizumab with bevacizumab (18). They found that ICI + TKI combinations tended to have a higher likelihood of providing maximal OS and PFS regardless of the IMDC risk group. Similarly, one meta-analysis performed by Riaz et al. showed that the combination of cabozantinib with nivolumab was associated with the highest rates of objective response and longer PFS and OS (19). However, the combination of Ipi + Nivo was associated with higher rates of complete response.

In our study, the observed superior rwTTNT in patients receiving ICI + TKI compared to Ipi + Nivo is consistent with what was observed in clinical trials. In CheckMate-214, the median PFS with Ipi + Nivo was 8.3 months, while in KEYNOTE-426, CheckMate 9ER, and CLEAR, the median PFS with ICI + TKI was 14, 15.6, and 22.1 months, respectively, in patients with intermediate- and poor-risk mccRCC (7, 8, 20, 21) . Although PFS is better with ICI + TKI, Ipi + Nivo leads to durable responses in a significant number of patients. In the final survival analysis of the CheckMate-214 trial with 8 years of median follow-up, ORR was achieved in 180 (42%) patients with intermediate- or poor-risk disease compared to 177 (41.6%) patients in the initial analysis (7). The median duration of response in the final analysis was 82.8 months. The median OS for patients with intermediate- or poor-risk disease was 46.7 months, which is notably higher than the rwOS of 28 months for patients receiving Ipi + Nivo reported in this study (22). Similarly, in the latest update to the CheckMate 9ER trial, patients treated with cabozantinib + nivolumab had a median OS of 49.5 months compared to a rwOS of 25 months observed in the ICI + TKI cohort in this study (20).

The reason for longer survival seen in clinical trials compared to shorter survival in our real-world patients is likely due to inherent bias present in clinical trials, such as the exclusion of patients with aggressive disease, including brain metastasis and comorbidities. A recent retrospective study found that patients with mRCC treated with TKIs in real-world settings were more likely to have poor-risk disease than those included in phase III clinical trials (23). In this study, 13% of patients in the Ipi + Nivo cohort and 19% in the ICI + TKI cohort had a confirmed Eastern Cooperative Oncology Group (ECOG) performance status of ≥ 2.

Considerable efforts have been made to develop biomarkers that distinguish therapeutic responses to ICI-based combinations in mRCC. Tumor mutational burden (TMB) and PD-L1 status have been shown to clinically correlate with better ICI outcomes in diverse malignancies (24). However, in the context of mRCC, studies have failed to demonstrate a clear relationship between clinical response and TMB or programmed death (PD)-L1 status (25). Although PD-L1 alone is not a reliable biomarker, sarcomatoid histology, which has higher PD-L1 positivity rates, was proven to have a superior response to ICI-containing regimens compared to VEGF-TKI monotherapy in mRCC (26, 27). Nevertheless, given that all first-line regimens contain an ICI, selecting Ipi + Nivo versus ICI + TKI based on histology alone remains challenging.

In recent studies, variation in ribonucleic acid (RNA) expression has emerged as a compelling predictive biomarker. A transcriptomic analysis of tumors from mRCC revealed seven molecular clusters with distinct therapeutic responses (28). ICI + bevacizumab and sunitinib had similar outcomes in clusters with angiogenesis-enriched mutations, whereas in angiogenesis-poor and immune-rich clusters, sunitinib had inferior performance compared to ICI-containing regimens. Similarly, in the phase 2 BIONIKK (a biomarker-driven, open-label, noncomparative, randomized) trial, which stratified patients into four groups based on distinct gene expression patterns, median PFS was longer with TKI alone in the pro-angiogenic group compared to Ipi + Nivo (29). Given that favorable-risk patients have higher rates of angiogenesis-enriched signatures, they may be better candidates for VEGF-TKI-containing regimens. This conclusion was aligned with the initial reports of Checkmate-214 in which Ipi + Nivo was outperformed by sunitinib in favorable-risk patients while ICI + TKI had improved outcomes across all risk groups. However, the final OS analysis from the CheckMate-214 trial demonstrated a trend toward improved OS with Ipi + Nivo compared to sunitinib in patients with favorable-risk disease (22). Thus, the predictive value of transcriptomics in estimating therapeutic response for mRCC remains unclear and complex.

Consequently, the decision between selecting Ipi + Nivo versus ICI + TKI centers on shared decision-making, weighing disease burden, treatment toxicities, and patient goals. In the absence of trials directly comparing these frontline therapies and with the lack of strong biomarkers, real-world studies are crucial for providing evidence to help clinicians with ICI-based combination selection and patient counseling.

Our study is the largest real-world study that directly compares these first-line therapies in a homogenous population of patients with mccRCC and intermediate or poor IMDC risk. Our results align with prior data showing better rwTTNT with ICI + TKI combinations compared to Ipi + Nivo with no statistically significant difference in rwOS. rwTTNT provides clinically relevant guidance regarding the duration of clinical benefit by encompassing both treatment efficacy and patient tolerance and compliance. A systematic review of phase II and III studies in advanced solid tumors found that time to subsequent therapy or death correlates to PFS (median R^2^ = 0.88) (30). Another study investigating time to next therapy, time to treatment failure, and objective response as intermediate endpoints for OS in mRCC found that time to next therapy had the strongest association with OS (31). In mccRCC, lengthening the time to next therapy is an important consideration in patients who are less likely to receive 2L therapy such as those with rapidly progressing disease or visceral metastases.

We acknowledge several limitations of our study, including its retrospective nature, residual confounding, baseline differences between cohorts, unavailability of objective response rates, the absence of data regarding treatment-related adverse events, and reasons for discontinuation as well as information on corticosteroid use and patient comorbidities. Additionally, we acknowledge the inherent limitations of rwTTNT as a metric for duration of clinical response, including its susceptibility to patient and prescriber preferences in treatment selection, as well as its inability to detect treatment-free intervals.

## Conclusion

In this large real-world study, patients with intermediate or poor IMDC risk mccRCC treated with ICI and TKI had superior rwTTNT compared to patients receiving Ipi + Nivo while rwOS was similar between both groups. In the absence of head-to-head clinical trials comparing first-line ICI-based combinations or predictive biomarkers, these findings offer real-world data on survival outcomes associated with Ipi + Nivo versus ICI + TKI and help guide clinicians in their decisions and patient counseling.
